# Tocilizumab but not Siltuximab prevents systemic inflammation in a humanized mouse model

**DOI:** 10.3389/fimmu.2026.1813955

**Published:** 2026-06-05

**Authors:** Liang Zhang, Jacqueline Wax, Torsten Goldmann, Afsaneh Mehrpouyan, Antje Müller, Frank Petersen, Gabriela Riemekasten, Xinhua Yu

**Affiliations:** 1 Research Center Borstel, Leibniz Lung Center, Priority Research Area Chronic Lung Diseases, Borstel, Germany; 2 Airway Research Center North, Member of the German Center for Lung Research (DZL), Großhansdorf, Germany; 3 Histology, Research Center Borstel, Leibniz Lung Center, Borstel, Germany; 4 Department of Rheumatology, University of Lübeck, Lübeck, Germany

**Keywords:** humanized mouse model, Siltuximab, systemic sclerosis, therapeutic efficacy, Tocilizumab, systemic inflammation

## Abstract

**Objectives:**

The present study aimed to assess the therapeutic efficacy of neutralizing monoclonal antibodies targeting a prominent proinflammatory cytokine in a PBMC transfer-induced humanized mouse model of systemic inflammation.

**Methods:**

Inflammatory cytokines were measured in human and murine sera using the LEGENDplex™ cytokine panel. Humanized mice were treated with neutralizing antibodies against human IL-6 (Siltuximab) or the human IL-6 receptor (Tocilizumab), along with matched IgG isotype controls.

**Results:**

Cytokine responses in the humanized mouse model were predominantly of human, not murine, origin. Elevated levels of human IL-6 were observed in both SSc patients and their corresponding mouse models. Preventive administration of Tocilizumab reduced anti-nuclear antibody production and mitigated disease severity in the PBMCs-transfer-induced humanized mouse model. In contrast, treatment with Siltuximab, an antibody targeting human IL-6, did not prevent disease development in the humanized mouse model. The lack of efficacy of Siltuximab was associated with the accumulation of human IL-6/anti-human IL-6 monoclonal antibody immune complexes.

**Conclusion:**

These findings highlight the pivotal role of IL-6 signaling in the SSc related systemic inflammation within the humanized mouse model and underscore the therapeutic potential of IL-6 receptor blockade. Furthermore, the PBMCs-based humanized mouse model offers a valuable preclinical platform for evaluating human-specific therapeutic interventions in systemic inflammation.

## Introduction

Systemic chronic inflammation (SCI) arises from sustained activation of the immune system and persistent release of pro-inflammatory cytokines, contributing to a wide range of pathological conditions and increased mortality (1). SCI can manifest as a chronic, low-grade inflammatory state triggered by multiple risk factors including microbiome dysbiosis, diet, obesity, lifestyle, and social and physical stress, exposure to xenobiotic, and sleep disturbances (2). In contrast, SCI may also be presented as a severe, high-grade inflammatory responses affecting multiple organs, which is frequently associated with severe infectious diseases as well as systemic autoinflammatory and autoimmune disorders (3, 4). For example, patients with systemic sclerosis (SSc), a chronic autoimmune rheumatic disease, frequently exhibit persistent inflammation involving multiple organs (5). Owning to the marked heterogeneity of its underlying causes, the pathogenesis of systemic chronic inflammation is highly complex.

Given the complexity and heterogeneity of systemic chronic inflammation in diseases, suitable experimental models are essential to unravel pathogenic mechanisms and test potential therapies. Mouse models have therefore been extensively used to investigate chronic inflammatory diseases (6–8). In case of SSc, more than twenty mouse models have been established, each of which recapitulates one or more hallmarks of SSc (9). Although humans and mice share substantial similarities as mammals, there are still considerable differences in the immune system and microenvironment between the two species, leading to a pathophysiological gap between mouse models and human disease (10, 11). In an effort to bridge this gap, some investigators have attempted to combine elements from both species, resulting in the creation of so-called humanized mouse models (12). Recently, our group has established a novel humanized mouse model by transferring peripheral blood mononuclear cells (PBMCs) from patients with SSc into *Rag2-/-/IL-2rg-/-* immunodeficient mice (13). Twelve weeks after the transfer, mice engrafted with PBMCs from SSc patients generated antinuclear antibodies (ANAs) mimicking the pattern of the respective donors and exhibited inflammatory infiltrates in multiple organs, including the lung, kidney, and liver. Owning to these features, this humanized SSc model represents a powerful experimental system for investigating systemic chronic inflammation and serves as a valuable platform for evaluating novel therapeutic interventions.

In a subsequent study, we demonstrated that both human T cells and B cells are indispensable for development of systemic chronic inflammation in this humanized mouse model (14). In the present study, we investigate molecular mechanisms underlying systemic inflammation. Cytokines are central regulators of inflammatory processes, and several cytokines—including interleukin-1β (IL-1β), IL-6, and tumor necrosis factor-α (TNF-α)—serve as established biomarkers of inflammatory activity (15, 16). However, their direct pathogenic roles in the development of systemic inflammation are likely context-dependent and remain elucidative. Here, we utilized this model to assess the contribution of cytokines in the development of systemic inflammation and evaluate therapeutic efficacy of neutralizing monoclonal antibodies targeting human cytokines, with a particular focus on IL-6.

## Materials and methods

### Patients and healthy subjects

Ten patients diagnosed with SSc were enrolled for the establishment of the PBMCs transfer-induced humanized mouse model. Additionally, 25 SSc patients were recruited to determine the serum levels of cytokines. All participants were sourced from the Department of Rheumatology at the University of Luebeck, Germany, and were diagnosed according to the 2013 ACR/EULAR criteria. Concurrently, 26 healthy donors were enrolled at the Research Center Borstel, Germany.

### Mice

Female RAG2 and IL-2 receptor gamma chain double knockout (C57BL/6NTac.Cg-*Rag2tm1Fwa Il2rgtm1Wjl*) mice, aged 8–12 weeks and having reached physiological and immunological maturity, were purchased from Taconic Biosciences, NY, USA. Mice were housed in individual ventilated cages (3 mice/cage) under specific pathogen free and biosafety level 2 conditions with a 12-hour light-dark cycle in the animal facility of the Research Center Borstel. Food and water were provided ad libitum. All animals were allowed to acclimatize for two weeks prior to experimentation.

### Ethics

All animal studies were reviewed and approved by the Animal Research Ethics Board of the Ministry for Energy Transition, Agriculture, Environment, Nature and Digitalization, Kiel, Germany (V241–50577/2017 (108-8/17)). All human doners in this study was conducted in accordance with the 1964 Declaration of Helsinki and its subsequent amendments or similar ethical standards. Approval for this study was obtained from the institutional ethics committee of the University of Luebeck (Az-113-2023), and written informed consent was obtained from all individual participants.

### Isolation and transplantation of human PBMCs

PBMCs were isolated from peripheral blood via density gradient centrifugation using Ficoll as previously described (13) and were cryopreserved in liquid nitrogen. In total, 10 patients, a sample sized estimated according to our previous study (14), were recruited as PBMC donors. On the day of the experiment, the frozen cells were rapidly thawed in a 37 °C water bath. The PBMCs were then transferred into fresh RPMI 1640 medium. After a single wash with RPMI 1640 medium, the cells were counted and suspended in RPMI 1640 medium at concentrations ranging from 1.5 to 3.3 × 10^7^ cells. For PBMC transfer, cells from each patient were equally administered to three *Rag2-/-/IL2rg-/-* mice housed in one cage by intraperitoneal injection. Following transplantation, each group of three mice that received PBMCs from a single patient was subjected to treatment with either human IgG1 isotype control, anti-hIL-6 monoclonal antibody (Siltuximab), or anti-hIL-6 receptor monoclonal antibody (Tocilizumab). Following previously established treatment protocols in humanized mouse models, with slight modifications (17, 18), the treatment regimen consisted of weekly injections of 0.2 mg of monoclonal antibody or IgG1 isotype control, commencing on day 1 following PBMC transfer. Five weeks after cell transfer, peripheral blood samples were collected to determine cytokine levels, aiming to assess immunological features during the early phase of disease development. Eleven weeks post- transfer, all mice were euthanized using an overdose of anesthesia to avoid potential alternations to the lung caused by CO_2_ exposure, and peripheral blood and tissues were harvested for the assessment of disease characteristics.

### Calculation of pathogenicity index

To assess the therapeutic efficacy of Tocilizumab and Siltuximab, a pathogenicity index as a measure of disease severity was calculated for each mouse using specific criteria outlined in **Supplementary Table 1**. These criteria assigned scores based on levels of ANA and the extent of infiltration area of human and murine immune cells in the lung, kidney, liver, and heart, with a maximum score of 20 points for each parameter. All above mentioned parameters were evaluated and scored blindly. The pathogenicity index for a mouse was determined by summing the scores for the five parameters, with a maximum possible score of 100 points. Mice that died or were sacrificed due to severe disease were assigned a score of 100 points directly.

### Flow cytometric analysis

Flow cytometric analysis was conducted to assess human lymphocytes in human PBMC and murine peripheral blood as described previously (13). Briefly, 1×10^6^ cells from each sample were stained with cellular markers containing an antibody mixture composed of the following fluorescent chromes-conjugated antibodies (all purchased from BioLegend, CA, USA): BV421-mouse-anti-human CD3 (Clone: UCHT1, Cat#300434), BV650-mouse-anti-human CD4 (Clone: RPA-T4, Cat#300536), APC-mouse-anti-human CD8 (Clone: SK1, Cat#980904), PerCP/cy5.5-mouse-anti-human CD20 (Clone: 2H7, Cat#302326), and FITC-mouse-anti-human CD45 (Clone: 2D1, Cat# 368508). For control staining, cells were stained with a mixture of isotype controls (all purchased from BioLegend) composed of BV421-Mouse IgG1 (Clone: MOPC-21, Cat#400158), BV650-Mouse IgG1 (Clone: MOPC-21, Cat# 400164), APC-Mouse IgG1 (Clone: MOPC-21, Cat#400120), PerCP/cy5.5-Mouse IgG2B (Clone: MPC-11, Cat#400338), and FITC-Mouse IgG1 (Clone: MOPC-21, Cat# 400108). After incubation, cells were washed twice with 2 ml FACS buffer, resuspended in 100 μL FACS buffer, then fixed by adding 50 μL 4% paraformaldehyde (PFA) solution, and finally measured by LSR II flow cytometer. Data analysis was performed using FACS Express software (*De Novo* Software, USA, version 7) and FlowJo™ software (BD Life Sciences, USA, version 10.8).

### Determination of human and murine cytokines

LEGENDplex™ (BioLegend, CA, USA) kits were utilized to quantify serum or plasma levels of both human and murine cytokines. Specifically, the Human Proinflammatory Cytokines 13-plex kit was employed to assess the concentrations of human IL-1β, IFN-α, IFN-γ, TNF-α, IL-6, IL-8, IL-10, IL-12 (p70), IL-17A, IL-18, IL-23, and IL-33 in serum or plasma samples obtained from patients, healthy subjects, and humanized mice. Additionally, the Mouse Inflammation Panel 13-plex kit was utilized to determine the levels of murine IL-1α, IL-1β, IL-6, IL-10, IL-12p70, IL-17A, IL-23, IL-27, MCP-1, IFN-β, IFN-γ, TNF-α, and GM-CSF in humanized mice. All assays were performed following the manufacturer’s protocols.

### Determination of anti-nuclear antibodies

Anti-nuclear antibodies (ANA) were assessed using the IIFT-HEp-20–10 EUROPattern Kit (Euroimmun, SH, Germany, Cat# FC 1522-2010), employing immunofluorescence staining in accordance with the manufacturer’s protocol. Serum or plasma samples designated for the assay were diluted at a ratio of 1:10 prior to testing. Immunofluorescence images were captured using confocal microscopy. Subsequently, ANA levels were independently and blindly scored by three colleagues, the Cohen’s kappa and Krippendorff’s alpha values are calculated to assign the consistency of individual scores. Scores ranged from 0 to 4, with values of 0, 1, 2, 3, and 4 corresponding to negative, weakly positive, positive, strongly positive, and very strongly positive, respectively. The final scores for each sample were calculated as the median values obtained from the independent assessments.

### Cell culture and stimulation

The human monocytic cell line U-937 was cultured in RPMI 1640 medium (PAN-Biotech, BY, Germany, Cat# P04-18047) supplemented with 10% fetal calf serum (FCS) (Gibco, MA, USA, Cat#10270) and 1% penicillin-streptomycin (PS) (PAN-Biotech, Cat# P06-07700). Cells were seeded at a density of 2×10^6^ cells per well in a 12-well plate and cultured in a humidified atmosphere with 5% CO_2_ at 37 °C for 1 hour. Subsequently, the cells were treated with different concentrations (0, 0.1, 1, and 10 μg/mL) of human IgG isotype control, Siltuximab, and Tocilizumab for 30 minutes at 37 °C and 5% CO_2_. Following treatment, cells were stimulated with human IL-6 at a concentration of 100 ng/mL for 10 minutes. After stimulation, cells were harvested for further analysis.

### Western blotting

U-937 cells were lysed using RIPA buffer supplemented with Complete™ Protease Inhibitor (Sigma-Aldrich, MA, USA, Cat#11697498001) solution at a volume of 25 μL per million cells. Subsequently, 20 μg of total protein from the cell lysate, was loaded into wells of an SDS-PAGE gel, separated by electrophoresis, and transferred onto a PVDF membrane (Millipore, MA, USA),. The membrane was then blocked using 1× Roti-ImmunoBlock blocking buffer for 1 hour and subsequently incubated with primary antibodies, including STAT3 antibody (CST, MA, USA, Clone: D3Z2G, Cat#12640S, 1:1000), anti-p-STAT3 Tyr 705 antibody (Santa Cruz, TX, USA, Clone: B-7, Cat#sc-8059, 1:200), or anti-β-actin antibody (CST, Clone: 13E5, Cat#4970, 1:5000), for 1 hour at room temperature. After washing three times with PBS-T (PBS-D + 0.05% Tween-20), the membrane was further incubated with secondary antibodies, including goat anti-mouse IgG(H+L) Alexa Fluor 680 conjugate secondary antibody (Thermo Fisher, MA, USA, Cat# A-21057, 1:5000) and goat anti-Rabbit IRDye800 secondary antibody (Biotrend, NRW, Germany, Cat# 611-132-122, 1:5000), for 1 hour at room temperature. Following another round of washing, the membrane was air-dried overnight, and protein bands were visualized using the LI-COR imaging system.

### Histological evaluation

The organs and tissues, including the lung, liver, kidney, heart, and spleen, were harvested from the mice and fixed in 4% paraformaldehyde overnight. After dehydration and paraffin embedding, the samples were sectioned into 4 μm thick slices for histological examination, sections were stained with hematoxylin and eosin (H&E) staining.

### Immunohistochemistry

Immunohistochemistry staining was conducted on murine tissue sections to assess the cellularity of infiltrated cells. Lung, kidney, heart, liver, and spleen paraffin-embedded sections underwent deparaffinization in xylene followed by rehydration in a gradient of ethanol. Subsequently, antigen retrieval was achieved by microwave heating with 1× citrate buffer (pH=6) (ZytoMed, BE, Germany, Cat#ZUC028-500), and sections were blocked using 3% H_2_O_2_ and a blocking buffer. They were then incubated with specific primary antibodies for 1 hour at room temperature, including rabbit anti-hCD45 (CST, Cat#13917, 1:500), rabbit anti-hCD20 (Invitrogen, MA, USA, Cat# PA5-16701, 1:500), rabbit anti-hCD3 (ZytoMed, Cat# RBK024-05, 1:100), or rat anti-mouse neutrophil antibodies (Cedarlane, ON, Canada, Cat# CL8993AP, 1:800). Following washing steps, the sections were treated with ImmPRESS-HRP goat anti-rat IgG polymer kit (VECTOR, Cat# MP-7404-50) or ZytoChem Plus-HRP polymer kit (ZytoMed, Cat#POLHRP-100), followed by incubation with 3-Amino-9-ethylcarbazole (AEC) (ZytoMed, Cat#ZUC054-200) to visualize immunoreactivity. Finally, counterstaining with hematoxylin was performed, and bright-field microscopy was used to capture images.

### Determination of IL-6-anti-IL-6 IgG immune complexes

A specialized specific flow cytometric assay was developed to quantify circulating hIL-6/anti-hIL-6 IgG immune complexes (ICs). Initially, 25 μL of assay buffer was dispensed into the wells of a 96-well V-bottom plate, followed by the addition of 25 μL of hIL-6 capture beads (BioLegend, Cat#740124), 25 μL of murine serum or plasma samples (1:10), and 25 μL of Alexa Fluor 488 anti-human IgG Fc recombinant antibody (BioLegend, Clone: QA19A42, Cat#366921, 1:25). For control staining, beads were incubated with Alexa Fluor 488 isotype mouse IgG1 (BioLegend, Clone: MOPC-21, Cat#400129). The plate was then incubated for 2 hours at room temperature on a shaker. Following incubation, the plate was washed twice, and the beads were resuspended in FACS buffer before being analyzed using a FACSymphony A1 flow cytometer within 3 days.

### Statistical analysis

All quantitative data are presented as median with interquartile range (Q1 (first quartile) – Q3 (third quartile)), while categorical data are expressed as number and percentage. Normal distribution of quantitative variables was assessed using the Shapiro-Wilk test or Kolmogorov-Smirnov test. Unpaired Student’s t-test or Mann-Whitney U test was employed for unpaired variables with and without normal distribution, respectively. Paired t-test or Wilcoxon matched-pairs signed rank test was used for the comparison of paired data. Friedman test was utilized for paired comparisons of different groups that do not follow the abnormal distribution. For categorical variables, statistical analysis was performed using Fisher’s exact test. Correlation analysis was conducted using the Spearman method, and P-values were adjusted using the Benjamini-Hochberg (BH) method for multiple comparisons. A P-value of < 0.05 was considered statistically significant.

## Results

### Human IL-6 is linked to signs of SSc in both the humanized mouse model and patients

**Figure 1:**
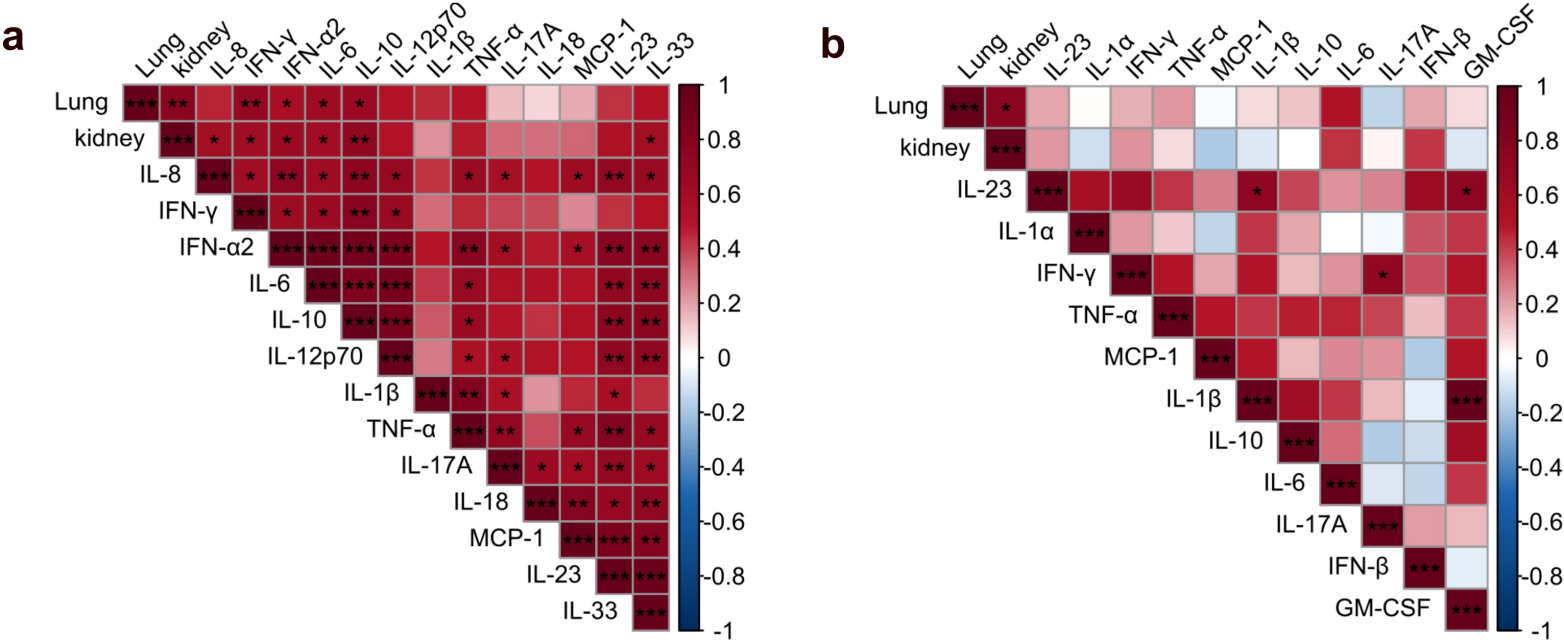
Human rather than murine derived cytokines correlated with severity of inflammation in of the humanized mouse model. This analysis indicates the correlation between cytokines levels and severity of tissue inflammation in mouse receiving PBMCs derived from patients with SSc to reveal disorder development related cytokines. The panel **(a)** shows associations between human cytokines and signs of lung or kidney inflammation in the humanized SSc model, while the panel **(b)** shows the correlation matrix for signs of tissue inflammation and murine cytokines. Spearman’s correlation coefficients are color-coded, with positive correlations depicted in red and negative correlations in blue, the murine IL-27 and IL-12p70 whose values are 0 in all individuals of SSc group, making the correlation analysis not applicable, therefore, they are removed from analysis P values in correlation analyses were adjusted using the Benjamini-Hochberg (BH) method for multiple comparisons. *P < 0.05, **P < 0.01, and ***P < 0.001.

To assess associations between a number of cytokines and experimental disease in the humanized mouse model, serum levels of 13 human cytokines, namely IL-1β, IFN-α2, IFN-γ, TNF-α, MCP-1, IL-6, IL-8, IL-10, IL-12p70, IL-17A, IL-18, IL-23, and IL-33, were determined in mice receiving PBMCs from SSc patients (n=16) and controls which received PBMC from healthy subjects (n=12). Serum samples were collected in previous experiments either at week 12 after PBMC transfer or after the mice died or were sacrificed due to the disease (13, 14). As shown in panel 1 of **Table 1** and **Supplementary Figure 1**, serum levels of human IL-6, IL-8, and IL-12p70 were elevated in mice receiving PBMC from SSc patients compared to those engrafted with PBMC from healthy controls. Conversely, no differences were observed for the levels of the other 10 cytokines between both groups. Despite lacking an adaptive immune system, *Rag2-/-IL-2rg-/-* mice possess the capacity to produce cytokines through innate immune cells. To ascertain whether murine cytokines contribute to disease development in the humanized mouse model, serum levels of 13 murine cytokines (9 overlapping with the human panel) were evaluated in the aforementioned groups of mice. Among the 13 murine cytokines assessed, none of cytokines exhibited differences between the two groups of mice (panel 2 of **Table 1** and **Supplementary Figure 1**). To further explore relationships between inflammatory cytokines and disease development in the humanized mouse model, we calculated correlations between levels of both human and murine cytokines and the severity of inflammation in the organs, which represented by the percentage of inflammatory area. Serum levels of four human cytokines—IFN-α2, IFN-γ, IL-6, and IL-10—exhibited positive correlations with the severity of inflammation both in the lung and the kidney (**Figure 1a**). In contrast, none of the murine cytokines displayed correlations with the severity of inflammation in either the lung or the kidney (**Figure 1b**). Taken together, these findings suggest that human, rather than murine cytokines, are intricately linked to disease development in the humanized mouse model of SSc.

**Table 1:** Serum levels of human and murine cytokines in the humanized mouse model.

Panel 1. Levels of human cytokines			
Cytokines (pg/mL)	Mice grafted with HD-PBMC (N = 12)	Mice grafted with SSc-PBMC (N = 16)	*P* values
IL-1β	0.00 (0.00-0.00)	0.00 (0.00-2.02)	0.137
IFN-α2	0.19 (0.00-0.53)	2.82 (0.00-23.05)	0.095
IFN-γ	602.36 (111.28-1196.91)	3183.91 (416.17-8133.87)	0.219
TNF-α	0.00 (0.00-0.00)	0.00 (0.00-1.10)	0.722
MCP-1	0.00 (0.00-0.45)	0.00 (0.00-4.50)	0.600
IL-6	0.00 (0.00-0.55)	5.66 (0.00-150.48)	0.036
IL-8	0.80 (0.41-1.77)	39.16 (1.58-108.20)	0.021
IL-10	1.26 (0.00-4.01)	79.85 (0.00-667.58)	0.077
IL-12p70	0.00 (0.00-0.33)	2.68 (0.00-16.95)	0.037
IL-17A	0.00 (0.00-0.00)	0.00 (0.00-0.00)	0.697
IL-18	0.00 (0.00-0.00)	0.00 (0.00-0.82)	0.073
IL-23	0.00 (0.00-0.00)	0.00 (0.00-1.83)	0.583
IL-33	0.00 (0.00-0.00)	0.00 (0.00-31.42)	0.314

Panel 2. Levels of murine cytokines			
Cytokines (pg/mL)	Mice grafted with HD-PBMC^$^ (N = 11)	Mice grafted with SSc-PBMC (N = 16)	*P* values
GM-CSF	0.00 (0.00-0.00)	0.00 (0.00-0.00)	0.451
IFN-β	0.00 (0.00-0.00)	0.00 (0.00-0.00)	0.451
IFN-γ	0.00 (0.00-0.00)	0.00 (0.00-0.90)	0.164
IL-1α	1.46 (0.75-4.95)	4.66 (2.14-9.83)	0.131
IL-1β	0.00 (0.00-0.00)	0.00 (0.00-0.00)	0.871
IL-6	2.35 (0.79-7.56)	13.08 (3.80-42.50)	0.144
IL-10	0.00 (0.00-0.00)	0.00 (0.00-0.00)	0.604
IL-12p70	0.00 (0.00-0.00)	0.00 (0.00-0.00)	0.258
IL-27	0.00 (0.00-0.00)	0.00 (0.00-0.00)	NA^#^
IL-17A	0.00 (0.00-0.41)	0.21 (0.00-0.45)	0.328
IL-23	0.00 (0.00-0.00)	0.00 (0.00-0.00)	0.254
MCP-1	0.00 (0.00-21.07)	0.00 (0.00-36.63)	0.927
TNF-α	1.41 (0.00-2.40)	2.79 (1.28-7.57)	0.169

^$^ One serum sample was not available for the measurement. Cytokines were determined in the sera of mice collected at week12 after PBMC transfer or on the day the mouse died or was sacrificed due to disease. Data were presented as Median (Q1-Q3). ^#^The levels of cytokines in all the individuals in SSc and HD groups are 0, so the comparison is not appliable.

To examine whether human patients exhibit a cytokine pattern similar to that observed in the humanized mouse model, serum levels of the 13 human cytokines were examined in patients diagnosed with SSc (n=25) and gender-matched healthy individuals (n=26). Due to challenges in recruiting healthy donors over the age of 60, the median age of SSc patients was approximately 6 years older than that of healthy controls. The analysis revealed that 8 of 13 cytokines were elevated in serum of patients with SSc compared to healthy donors (**Supplementary Table 2**). These included IL-1β, IFN-α2, MCP-1, IL-6, IL-10, IL-12p70, IL-17A, and IL-23, whereas the remaining 5 cytokines were not different from healthy controls.

When comparing the serum cytokine values between the humanized mouse model and the human *ex vivo* data, IL-6, most likely derived from human PBMCs, emerged as prominent cytokines associated with features of SSc. Because of our findings and due to the fact that IL-6 has already been a therapeutic target in SSc, albeit with limited success, we chose to further examine the efficacy of both Tocilizumab and Siltuximab in our humanized mouse model at early disease phase.

### Treatment with Tocilizumab but not Siltuximab shows therapeutic efficacy

**Figure 2:**
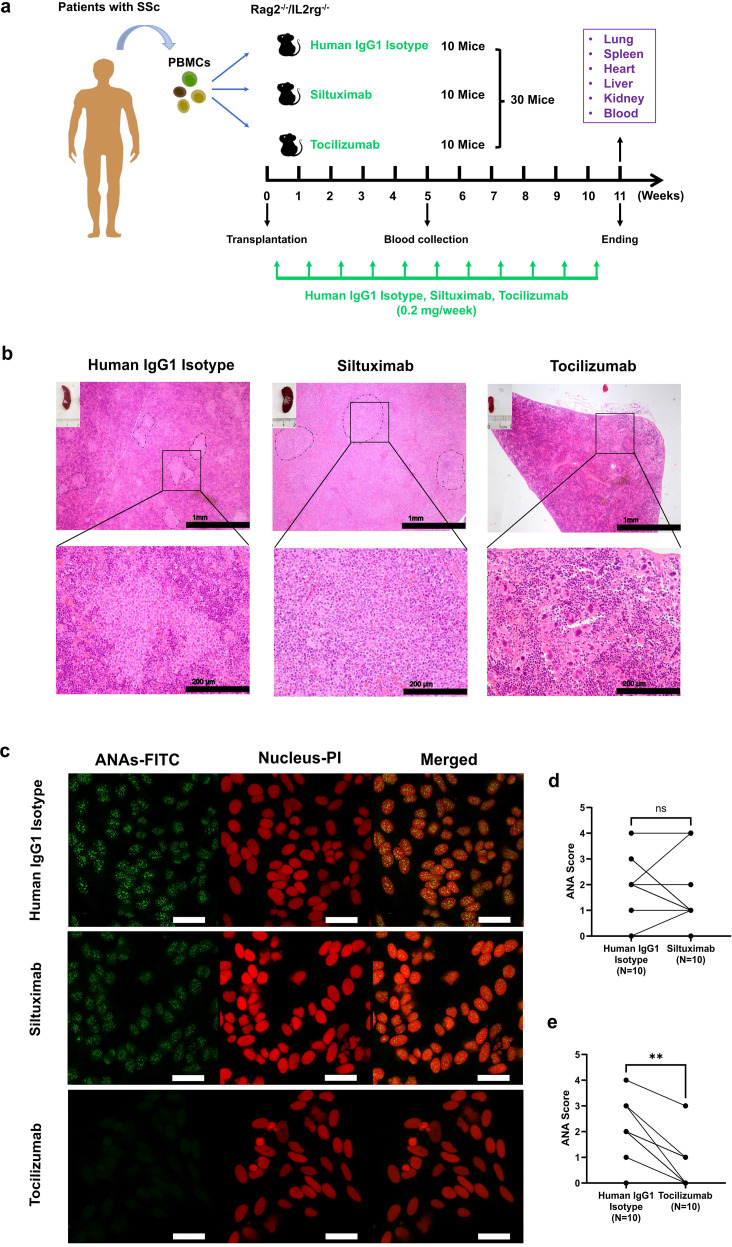
Effect of Tocilizumab and Siltuximab on restoration of spleen white pulp and on anti-nuclear antibody generation in the humanized mouse model. **(a)** Schematic overview of the experimental design. **(b)** Representative graphs photos of spleen and micrographs of hematoxylin-eosin (H&E) staining of spleen sections from mice receiving SSc PBMC and treated with IgG1 isotype control, Tocilizumab, or Siltuximab. The dotted line indicates the well-structured white pulp. Scale bars = 1 mm (low magnification, upper panel) and 200 μm (high magnification, lower panel). **(c)**. Representative confocal microscopy images (63x) depicting antinuclear antibody (ANA) presence in recipient mice, Scale bars = 50 μm. Comparison of ANA scores between mice treated with IgG1 isotype control and those treated with Siltuximab **(d)**, as well as between recipient mice treated with IgG1 isotype control and those treated with Tocilizumab **(e)**. Statistical significance of these comparisons was determined using the Wilcoxon matched-pairs signed rank test, ns: not significant, **P < 0.01.

Another subset of patients diagnosed with SSc was recruited as PBMC donors (n=10) for this experiment, comprising 6 individuals with dcSSc and 4 with lcSSc. The demographic and clinical characteristics of these SSc patients are summarized (**Supplementary Table 3**) including skin involvement and antibody specificity. As depicted in the experimental design (**Figure 2a**), 30 *Rag2-/-/IL-2rg-/-* mice used in this experiment were divided into three groups (each n=10). PBMCs isolated from each patient were transferred at an equal quantity into three *Rag2-/-/IL-2rg-/-* recipient mice, each of which came from the three groups. Prior to their transplantation into recipient mice, the cellular composition was assessed using flow cytometry with antibodies targeting human CD45, CD3, and CD20. The analysis revealed that approximately 70% of CD45^+^ PBMCs were T cells (CD45^+^CD3^+^), followed by CD45^+^CD3^-^CD20^-^ leukocytes constituting 20-30% of the population, and B cells (CD45^+^CD20^+^) making up approximately 5% of the total cells (**Supplementary Figure 2**). To assess the therapeutic efficacy of Tocilizumab and Siltuximab during early stages of disease development, a preventive strategy was utilized in this experiment. Subsequent to the PBMC transfer, starting from the 2nd day, these three groups of mice were subjected to weekly intraperitoneal (*i.p.*) injections of human IgG1 isotype control, Tocilizumab, or Siltuximab. In addition, the function of Tocilizumab and Siltuximab on blocking IL-6 signaling was validated *in vitro* (**Supplementary Figure 3**).

After engrafting the PBMCs, the presence of human PBMCs in murine spleen of each mouse was assessed by flow cytometric at the experimental endpoint. Analysis of splenocytes showed that human leukocytes were detectable in spleen of all recipient mouse (**Supplementary Figure 4**).

In addition, five mice died or had to be sacrificed due to severe disease before the end of the experiment. This included two mice from the group treated with IgG1 isotype control and three mice from the group treated with Siltuximab (**Supplementary Figure 5**). In contrast, none of the mice treated with Tocilizumab died or had to be sacrificed before the end of the experiment. However, the difference in survival rates among the groups did not reach statistical significance. Tissue samples from four out of the five deceased mice were accessible for histological evaluation, while spleen samples from three of these mice were available for flow cytometric analysis.

To evaluate the effect of the treatment, we first determined whether the transferred human PBMCs were translocated in the spleens of recipient mice. As expected from previous experiments (13, 14), *Rag2^-/-^/IL-2rg^-/-^* mice receiving SSc patient PBMC and being treated with human IgG1 isotype control showed an enlarged spleen with well-structured white pulps, indicating the presence of functional human lymphocytes (**Figure 2b**). The splenomegaly and restoration of the white pulp was also observed in mice treated with Siltuximab, but not in mice treated with Tocilizumab (**Figure 2b**).

Given that the presence of ANA is another immunological hallmark of the humanized mouse model (13, 14), we next determined levels of ANA in plasma of the three groups of mice using cell-based indirect immunofluorescence staining with confocal microscopy. The kappa values are calculated to indicate the consistency of ANA scores evaluated by three investigators blindly; the Cohen’s kappa values are all higher than 0.8 and Krippendorff’s alpha value is 0.798 (**Supplementary Table 4**). Representative micrographs in **Figure 2c** display the presence of ANA in mice treated with IgG1 isotype control or Siltuximab, but infrequently in mice treated with Tocilizumab. Blind scoring of the images indicated that levels of ANA were comparable between mice treated with IgG1 isotype control and those treated with Siltuximab (**Figure 2d**), whereas levels of ANA were significantly lower in mice treated with Tocilizumab than in those treated with IgG1 isotype control (*P* < 0.01) (**Figure 2e**).

**Figure 3:**
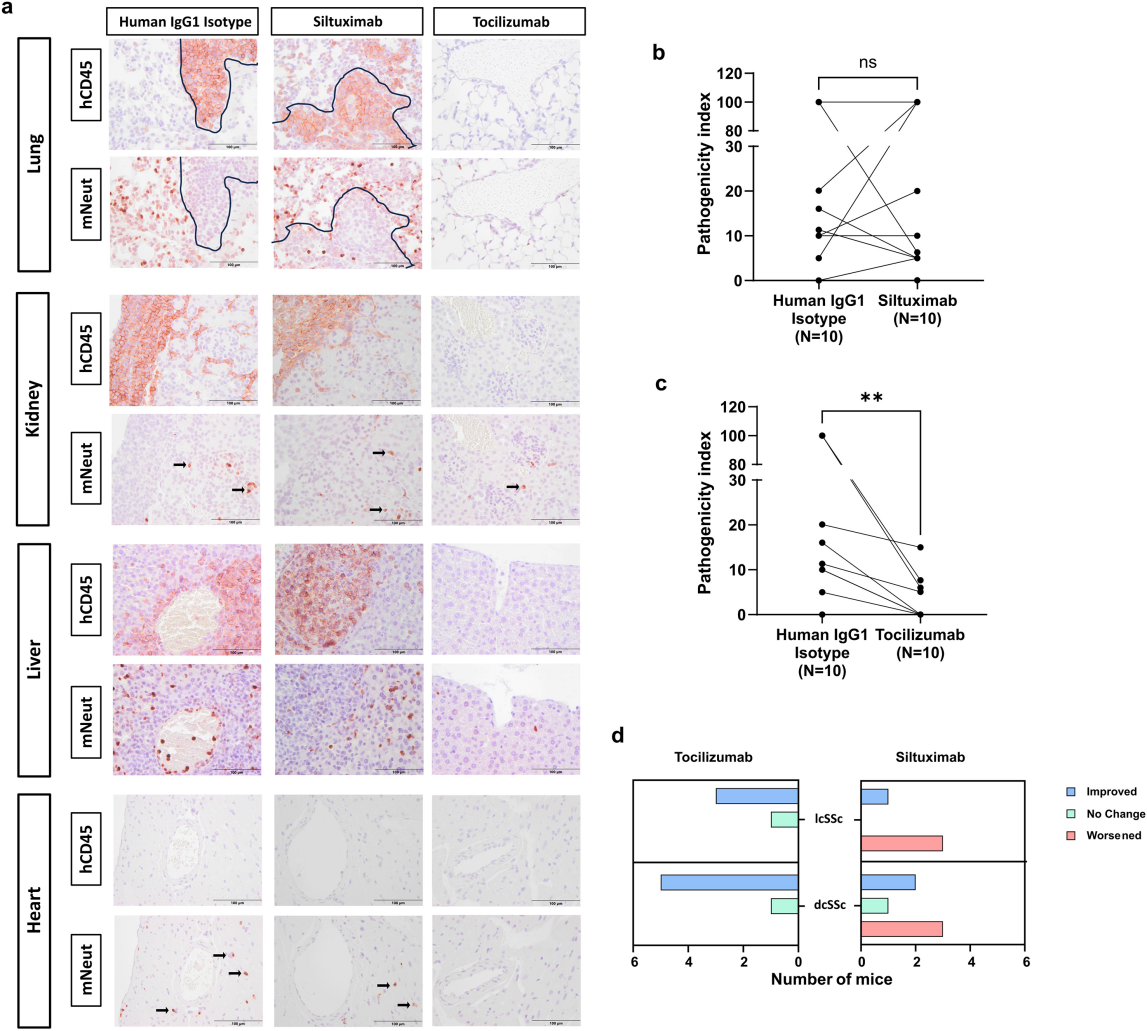
Effect of Tocilizumab and Siltuximab on tissue histopathology and disease severity in the humanized mouse model. **(a)** Immunohistochemistry staining using antibodies targeting hCD45 and mNeut were performed to assess the infiltration of human leukocytes and mouse neutrophils, respectively. Representative micrographs of are shown. Dotted lines delineate the areas of infiltration by human leukocytes and murine neutrophils in the lung, while black arrows indicate positively stained cells. Comparison of pathogenicity index (for index definition see **Supplementary Table 1**) between mice treated with IgG1 isotype control and mice treated with Siltuximab **(b)**, and between mice treated with IgG1 isotype control and mice treated with Tocilizumab **(c)**. Efficacy of Tocilizumab and Siltuximab in mice receiving PBMCs from patients with lcSSc or dcSSc **(d)**. Statistical significance of comparisons was determined using the Wilcoxon matched-pairs signed rank test. ns, not significant, **P < 0.01. Scale bars = 100 μm.

Next, we conducted assessments of tissue inflammation across the three groups of mice. Immunohistochemistry staining with a hCD45 antibody and an antibody against murine neutrophils were employed to visualize the infiltration into murine tissue (**Figure 3a**). As illustrated in **Figure 3a**, infiltration of both human leukocytes and murine neutrophils was evident in the lung, kidney, and liver of mice treated with human IgG1 isotype control or Siltuximab, while such infiltration was absent in mice treated with Tocilizumab. Notably, while inflammation in the lung, kidney, and liver was primarily driven by human leukocytes, heart inflammation was predominantly attributed to murine neutrophils (**Figure 3a**). Furthermore, human leukocytes and murine neutrophils exhibited distinct patterns of infiltration within the tissues, as indicated by their distribution in the lungs (**Figure 3a**).

To assess the therapeutic efficacy of Tocilizumab and Siltuximab, a pathogenicity index (**Supplementary Table 1**) was calculated for each mouse. As depicted in **Figure 3b**, the median pathogenicity index in mice treated with IgG1 isotype control was 10.68, similar to that observed in mice treated with Siltuximab (8.16) (*P* = 0.652). In contrast, the median pathogenicity index in mice treated with Tocilizumab was 0, significantly lower than that of mice treated with IgG1 isotype control (*P* < 0.01) (**Figure 3c**).

In previous clinical trials using Tocilizumab, only dcSSc patients were recruited, which leaves the question of efficacy of Tocilizumab for lcSSc patients unclear (19, 20). To evaluate potential efficiency of Tocilizumab in lcSSc patients, patients with lcSSc were recruited as donors of PBMCs (n=4), apart from dcSSc (n=6). When recipient mice were stratified into lcSSc and dcSSc based on the source of PBMCs, the analysis indicated that Tocilizumab treatment was effective for mice receiving PBMCs from both subtypes of SSc (**Figure 3d**).

### Inefficacy of Siltuximab treatment is associated with the accumulation of hIL-6-anti-hIL-6 IgG immune complexes

**Figure 4:**
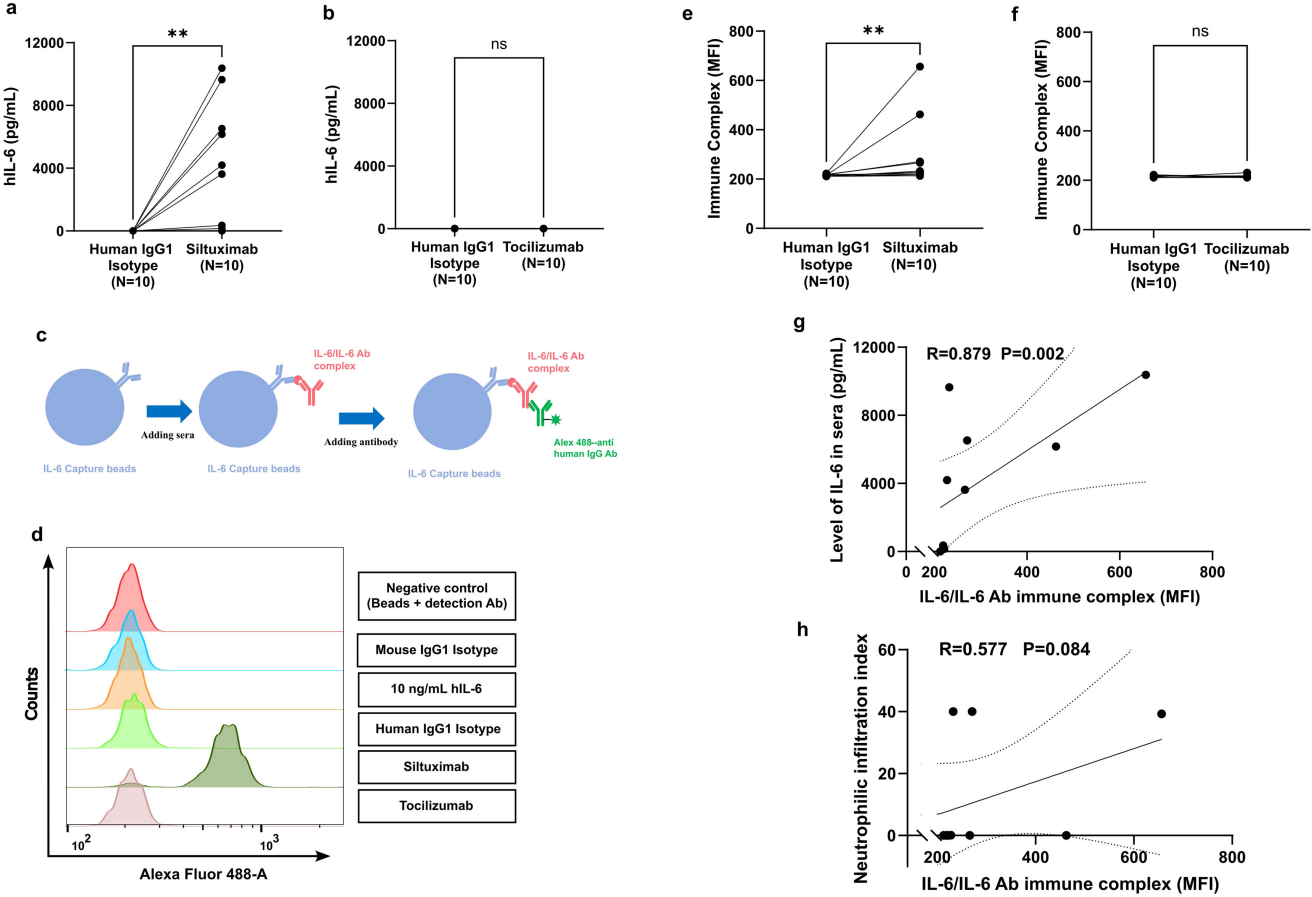
Association of treatment of Siltuximab with an accumulation of hIL-6-anti-hIL-6 IgG immune complexes (ICs). Comparison of circulating levels of IL-6 between Siltuximab-treated mice and IgG1 isotype control-treated mice **(a)**, and between Tocilizumab-treated mice and IgG1 isotype control-treated mice **(b)**. Schematic diagram illustrating the experimental design to measure hIL-6-anti-hIL-6 IgG ICs **(c)**. Representative histogram from flow cytometric analysis depicting the detection of hIL-6-anti-hIL-6 IgG ICs in plasma of mice treated with Siltuximab, Tocilizumab, or IgG1 isotype controls **(d)**. Comparison of levels of circulating hIL-6-anti-hIL-6 IgG ICs between mice treated with human IgG1 isotype control and those treated with Siltuximab **(e)**, and between mice treated with human IgG1 isotype control and those treated with Tocilizumab **(f)**. Correlation analysis between levels of hIL-6 and levels of hIL-6-anti-hIL-6 IgG ICs **(g)**. Correlation analysis between levels of the hIL-6-anti-hIL-6 IgG ICs and neutrophil infiltrations **(h)**. Spearman’s rank correlation coefficient (R) and associated P values are indicated. Statistical significance of comparisons was determined using the Wilcoxon matched-pairs signed rank test. ns, not significant, **P < 0.01, *P* values < 0.05 is considered as significance.

Considering that both Tocilizumab and Siltuximab effectively blocked human IL-6 signaling *in vitro* (**Supplementary Figure 3**), the notable contrast in their therapeutic efficacy *in vivo* prompted further experiments. To investigate potential reasons for the observed inefficacy of Siltuximab, we assessed its ability to properly neutralize human IL-6 *in vivo*. Unexpectedly, mice treated with Siltuximab exhibited extraordinarily high levels of circulating human IL-6. In contrast, the levels of circulating human IL-6 in mice treated with IgG1 isotype control were significantly lower than those in the Siltuximab treated group (**Figures 4a, b**, **Supplementary Table 5**).

We therefore speculate that the extraordinarily high levels of circulating IL-6 are not due to high expression of free IL-6. Given that immune complexes (ICs) exhibit much longer serum half-lives due to FcRn-mediated recycling (21), we hypothesize that the high levels of circulating IL-6 in Siltuximab-treated mice are due to an accumulation of hIL-6-anti-hIL-6 IgG ICs. To validate this hypothesis, a unique in-house flow cytometric experiment was designed to detect the hIL-6-anti-hIL-6 IgG ICs (**Figures 4c, d**). As expected, levels of circulating hIL-6-anti-hIL-6 IgG ICs in the Siltuximab-treated group were significantly higher than those in the human IgG1 isotype control-treated group (**Figure 4e**). Similar to the IgG1 isotype control-treated mice, all Tocilizumab-treated mice were negative for the presence of hIL-6-anti-hIL-6 IgG ICs (**Figure 4f**). Moreover, a strong positive correlation between levels of the ICs and levels of IL-6 detected using the Human Proinflammatory Cytokines 13-plex Kit was observed in mice treated with Siltuximab (**Figure 4g**), supporting the hypothesis that the extraordinarily high levels of circulating IL-6 detected in sera of Siltuximab-treated mice was due to an accumulation of hIL-6-anti-hIL-6 IgG ICs. Additionally, a trend to a positive correlation was observed between the levels of hIL-6-anti-IL-6 IgG ICs and the severity of murine neutrophilic infiltration in Siltuximab-treated mice (R = 0.577, *P* = 0.084) (**Figure 4h**).

## Discussion

Using a newly developed humanized mouse model (13, 14), this study investigated molecular pathomechanisms underlying systemic inflammation associated with SSc. Notably, levels of human IL-6 were found to be elevated in diseased mice. Moreover, levels of IL-6 correlated with disease severity which was assigned based on a composite score comprising organ infiltration by human and/or murine immune cells and appearance of ANA. Subsequently, we evaluated therapeutic efficacy of neutralizing monoclonal antibodies targeting IL-6 signaling on the development of systemic inflammation. The results demonstrated that Tocilizumab, but not Siltuximab, effectively prevented development of systemic inflammation in this humanized mouse model.

Tocilizumab, developed by Sato and colleagues (22), has gained regulatory approval for the treatment of various immune-related disorders, including rheumatoid arthritis, cytokine release syndrome, Castleman disease, and SSc-ILD (23). Two phase III clinical trials have revealed that Tocilizumab predominantly improves ILD in SSc patients, however, with limited efficacy observed for other disease manifestations (19, 20). This discrepancy in therapeutic outcomes may be attributed to the timing of treatment initiation, as patients enrolled in these trials typically exhibited skin involvement. Interestingly, the present study demonstrates that early administration of Tocilizumab, initiated prior to the onset of clinical symptoms, effectively prevents ANA generation of ANA and systemic inflammation in the humanized mouse model. Therefore, these findings provide evidence for an early therapeutic intervention of systemic inflammation, highlighting the potential benefits of initiating Tocilizumab treatment during the subclinical phase of inner organ involvement. In addition, Tocilizumab demonstrated notable efficacy in mice that received PBMCs from patients with both dcSSc and lcSSc, suggesting its potential efficacy across different subtypes of the disease.

In sharp contrast to Tocilizumab, Siltuximab failed to demonstrate therapeutic efficacy in the humanized mouse model of SSc, even exacerbating some disease manifestations. This unexpected outcome contrasts with the neutralizing effect of Siltuximab on IL-6 signaling of a monocyte-like cell observed *in vitro*. Notably, compared to humanized antibodies such as Tocilizumab, which exhibit 95% homology to human IgG, chimeric antibodies like Siltuximab display only 65% homology to human IgG (24). While this difference theoretically suggests a potentially higher immunogenicity for Siltuximab compared to Tocilizumab, empirical evidence indicates otherwise (25). Furthermore, the efficacy and safety of Siltuximab have been validated in clinical trials for Castleman’s disease (26), further dispelling concerns regarding its immunogenic potential.

Remarkably, mice treated with Siltuximab displayed a high amount of circulating IL-6-anti-IL-6 IgG ICs in peripheral blood. This accumulation may underlie the observed lack of therapeutic efficacy in the humanized mouse model of SSc. The formation and accumulation of ICs during monoclonal antibody therapy are common occurrences (27, 28). The efficacy of therapeutic antibodies relies on the subsequent interaction of ICs with the complement system and phagocytic cells (27). For example, Rituximab, which targets CD20 on B cells, induces B cell depletion via antibody-dependent cellular cytotoxicity and complement-dependent cytotoxicity upon the formation of CD20-anti-CD20 IgG ICs (29). However, proper clearance of ICs is crucial for immune homeostasis, as their accumulation can lead to pathogenic conditions and the production of proinflammatory cytokines, including IL-6 (30, 31). The clearance of circulating ICs typically involves monocytes and complement receptor type 1 on erythrocytes (32, 33). Importantly, the accumulation of hIL-6-anti-hIL6 IgG ICs has not been observed in Castleman’s disease patients treated with Siltuximab (26), suggesting efficient clearance mechanisms in humans. Based on our observations, we propose the following model to explain the accumulation of IL-6–anti–IL-6 IgG IC: Transfer of PBMCs from patients with SSc leads to increased levels of circulating free IL-6, as shown in the present study. This elevated IL-6 plays a critical role in disease pathogenesis, as evidenced by the observation that blockade of the IL-6 receptor with tocilizumab effectively prevents the development of systemic inflammation. In contrast, treatment with siltuximab results in the formation of IL-6–anti–IL-6 IgG immune complexes. These complexes appear to be inefficiently cleared by murine monocytes and erythrocytes, although the underlying mechanisms remain unclear. The accumulation of IC will in turn stimulate the production of IL-6. Consequently, continuously produced IL-6, driven by SSc PBMC transfer and the presence of IC (30, 31), is sequestered by the antibody, leading to the progressive accumulation of IL-6–anti–IL-6 IgG IC over time. This accumulation may lead to IC deposition in various tissues, subsequently mediating inflammation cell infiltration, particularly by neutrophils (34).

It is intriguing that the infiltration of murine neutrophils into multiple internal organs, including the lung, kidney, liver, and heart, was observed in the humanized mouse model. Since the infiltration of murine immune cells had not been previously determined in our previous studies (13, 14), the present study shows, for the first time, the involvement of murine neutrophils in the pathogenesis of the humanized mouse model. Interestingly, increased infiltration of murine neutrophils was evident in multiple internal organs of mice treated with Siltuximab or IgG1 isotype control, with a more pronounced extent in the former group. This finding suggests that the infiltration of murine neutrophils is a consequence of the transfer of PBMCs from SSc patients, which is further augmented by the accumulation of hIL-6-anti-hIL-6 IgG ICs. The role of murine neutrophils in this humanized mouse model is of paramount importance and warrants further investigation.

This study is subject to several limitations. First, to assess the therapeutic efficacy of Tocilizumab and Siltuximab, PBMCs were taken from only ten patients with SSc. This relatively small sample size may result in limited statistical power. The small cohort precluded stratified analysis to evaluate the relationship between donor SSc subtypes and disease severity or mortality in the mouse model. Second, cryopreserved PBMCs were employed in this study, the use of frozen PBMCs may impair their capacity for expansion and migration. Third, it cannot be ruled out that the elevated IL-6 level in the SSc cohort at least to a certain extent is due to the higher age of this group in comparison to the control group, since ageing goes along with increased amounts of IL-6 (35). Despite these limitations, the findings of this study offer compelling evidence regarding the therapeutic efficacy of Tocilizumab and Siltuximab in the humanized mouse model.

In summary, this study underscores the crucial role of human IL-6 in the pathogenesis of systemic inflammation within a humanized mouse model mimicking pathogenic features seen in SSc. It suggests that Tocilizumab, but not Siltuximab, represents an effective treatment for preventing autoimmunity and systemic tissue inflammation in this model. Moreover, this study serves as a notable example of utilizing a humanized mouse model to assess the efficacy of biological treatments targeting human molecules.

## Data Availability

The original contributions presented in the study are included in the article/**Supplementary Material**. Further inquiries can be directed to the corresponding authors.
