# Supplementary material for: Tocilizumab but not Siltuximab prevents systemic inflammation in a humanized mouse model
Source: Front Immunol. 2026 Jun 5;17:1813955. doi: 10.3389/fimmu.2026.1813955 (PMC13278986; doi:10.3389/fimmu.2026.1813955)
Supplement: Supplementary file 1 [file DataSheet1.pdf]

Supplementary information

Table S1. Criteria used for scoring pathogenicity index in the humanized mouse model

**Evaluation parameters**	**Description**	**Score**	**Maximal score ***
**ANA**	Negative (0)	0	20
	Weakly Positive (1)	5	
	Positive (2)	10	
	Strongly Positive (3)	15	
	Very Strongly Positive (4)	20	
**Lung inflammation**	Infiltration of human leukocytes	0-10	20
	Infiltration of murine neutrophils	0-10	
**Kidney inflammation**	Infiltration of human leukocytes	0-10	20
	Infiltration of murine neutrophils	0-10	
**Liver inflammation**	Infiltration of human leukocytes	0-10	20
	Infiltration of murine neutrophils	0-10	
**Heart inflammation**	Infiltration of human leukocytes	0-10	20
	Infiltration of murine neutrophils	0-10	
**Total**			100

*Mice died or had to be sacrificed due to severe disease were assigned a score of 100 directly. Score for tissue inflammation was estimated based on the percentage of infiltrated area.

Table S2. Comparison of clinical characterization and circulating cytokines between healthy subjects (HD) and patients with SSc

**Table S2a. Clinical characterization of HD and patients with SSc**			
	**HD (N=26)**	**SSc (N=25)**	**P value (SSc vs HD)**
**Age**	49.5 (40.25-53.25)	56 (45-63.5)	0.026
**Gender**			0.743
Female	19 (73.08%)	20 (80%)	
Male	7 (26.92%)	5 (20%)	
**Classification***			
lcSSc	/	15 (60%)	/
dcSSc	/	9 (36%)	/
**Autoantibodies**			
**ANAs**	/	25 (100%)	/
Scl-70	/	9 (36%)	/
RNA pol-III	/	1 (4%)	/
ACA	/	9 (36%)	/
Ro-52	/	10 (40%)	/
Ro/SS-A	/	2 (8%)	/
Pm/Scl	/	2 (8%)	/
**Non-ANAs**			
MDA-5	/	1 (4%)	/
AMA-M2	/	1 (4%)	/
AT_1R [&]	/	20 (87%)	/
ET_AR [&]	/	15 (65%)	/
**Organ/tissue involvement**			
Lung fibrosis	/	7 (28%)	/
PAH	/	3 (12%)	/
Heart	/	1 (4%)	/
Kidney	/	2 (8%)	/
Muscle	/	1 (4%)	/
Esophagus	/	3 (12%)	/
Gastro-intestinal	/	0 (0%)	/

**Table S2b. Circulating cytokines of HD and patients with SSc**			
	**HD (N=26)**	**SSc (N=25)**	**P value (SSc vs HD)**
**Cytokines** (pg/ml)			
IL-1β	9.52 (3.91-16.90)	20.46 (10.40-43.92)	0.015

IFN-α2	1.31 (0.00-3.46)	5.26 (1.64-15.34)	0.001
IFN-γ	6.56 (0.00-11.68)	11.68 (1.73-36.17)	0.066
TNF-α	6.57 (0.00-23.57)	13.75 (5.55-52.82)	0.079
MCP-1	375.6 (302.4-474.3)	469.2 (379.0-630.4)	0.033
IL-6	2.95 (0.00-5.58)	14.45 (3.01-30.89)	0.003
IL-8	27.36 (10.38-68.91)	22.54 (18.85-47.33)	0.446
IL-10	1.14 (0.86-3.05)	2.74 (1.05-4.96)	0.043
IL-12p70	2.11 (0.00-6.48)	9.47 (2.02-14.44)	0.017
IL-17A	1.87 (0.00-6.74)	11.25 (0.00-21.91)	0.024
IL-18	152.8 (134.3-204.9)	216.8 (143.9-276.0)	0.078
IL-23	0.00 (0.00-32.03)	99.77 (35.04-711.40)	<0.001
IL-33	216.20 (0.00-504.10)	218.00 (0.00-688.20)	0.601

Data were presented as Median (Q1-Q3). *Information of classification of one patient is not applicable.

& Information of AT1R and ETAR antibodies of two patients is not applicable

Table S3. Demographic and clinical characterization of SSc patients (n=10) used as PBMC donors for the humanized mouse model

	**SSc (N=10)**
**Age**	65(51-69)
**Gender**	
Female	4 (40%)
Male	6 (60%)
**Classification**	
lcSSc	4 (40%)
dcSSc	6 (60%)
**Autoantibodies**	
**ANAs #**	9 (100%) #
Scl-70	3 (30%)
RNA pol-III	3 (30%)
ACA	3 (30%)
Ro-52	1 (10%)
Ro/SS-A	1 (10%)
Pm/Scl	1 (10%)
**Non-ANAs**	
MDA-5	0 (0%)
AMA-M2	0 (0%)
AT_1R	0 (0%)
ET_AR	0 (0%)
**EUSTAR***	2.750 (0.875-4.875)
**Organ/tissue involvement**	
Lung fibrosis	6 (60%)
PAH	2 (20%)
Heart	2 (20%)
Kidney	1 (10%)
Muscle	0 (0%)
Esophagus	0 (0%)
Gastro-intestinal	0 (0%)

#Information of ANA of one patient is not applicable. * Information of EUSTAR of 4 patients are not applicable.

Table S4. Kappa values for consistency of ANA level scoring evaluation in humanized mouse model

Cohen Kappa Values (Individual comparisons)			
**Comparison**	**Weighted Kappa value**	**Z score**	**P value**
W1 vs W2	0.802	4.75	2.08×10^{-6}
W1 vs W3	0.832	5.01	5.34×10^{-7}
W2 vs W3	0.874	4.91	8.94×10^{-7}
**Krippendorff's alpha Values (Group comparison)**			
**Comparison**	**Weighted Kappa value**	**Z score**	**P value**
W1 vs W2 vs W3	0.798	NA	NA

W1= worker 1, W2= worker 2, W3= workers. The Krippendorff's alpha reflects magnitude of reliability rather than significant testing, therefore, Z score and P value are not applicable.

Table S5. Levels of circulating human-derived cytokines in sera of humanized mice treated with Tocilizumab, Siltuximab or IgG1 isotype control

Cytokines (pg/mL)	Human IgG1 (n=10)	Siltuximab (n=10)	Tocilizumab (n=10)
IL-1β	0 (0-0)	0 (0-0) ^{ns}	0 (0-0) ^{ns}
IFN-α2	0 (0-0)	0 (0-0) ^{ns}	0 (0-0) ^{ns}
IFN-γ	6.75 (0-251.0)	50.68 (0-300.6) ^{ns}	0 (0-40.5) ^{ns}
TNF-α	0 (0-0)	0 (0-0) ^{ns}	0 (0-0) ^{ns}
MCP-1	0 (0-0)	0 (0-0) ^{ns}	0 (0-0) ^{ns}
IL-6	0 (0-0)	3909 (121.3-7308) **	0 (0-0) ^{ns}
IL-8	0 (0-1.6)	0 (0-0) ^{ns}	0 (0-0) ^{ns}
IL-10	0 (0-0)	0 (0-0.54) ^{ns}	0 (0-0) ^{ns}
IL-12p70	0 (0-0)	0 (0-0) ^{ns}	0 (0-0) ^{ns}
IL-17A	0 (0-0)	0 (0-0) ^{ns}	0 (0-0) ^{ns}
IL-18	0 (0-0)	0 (0-0) ^{ns}	0 (0-0) ^{ns}
IL-23	0 (0-0)	0 (0-0) ^{ns}	0 (0-0) ^{ns}
IL-33	0 (0-0)	2359 (0-4306) ^{ns}	0 (0-0) ^{ns}

Cytokines were determined in plasma collected from mice at the 5th week post-PBMC transfer. Data are presented as median (Q1-Q3). Statistical significance was determined using Friedman test, all P values were calculated by comparing to the human IgG isotype control group. ns: not significant, ** P<0.01,

Panel 1

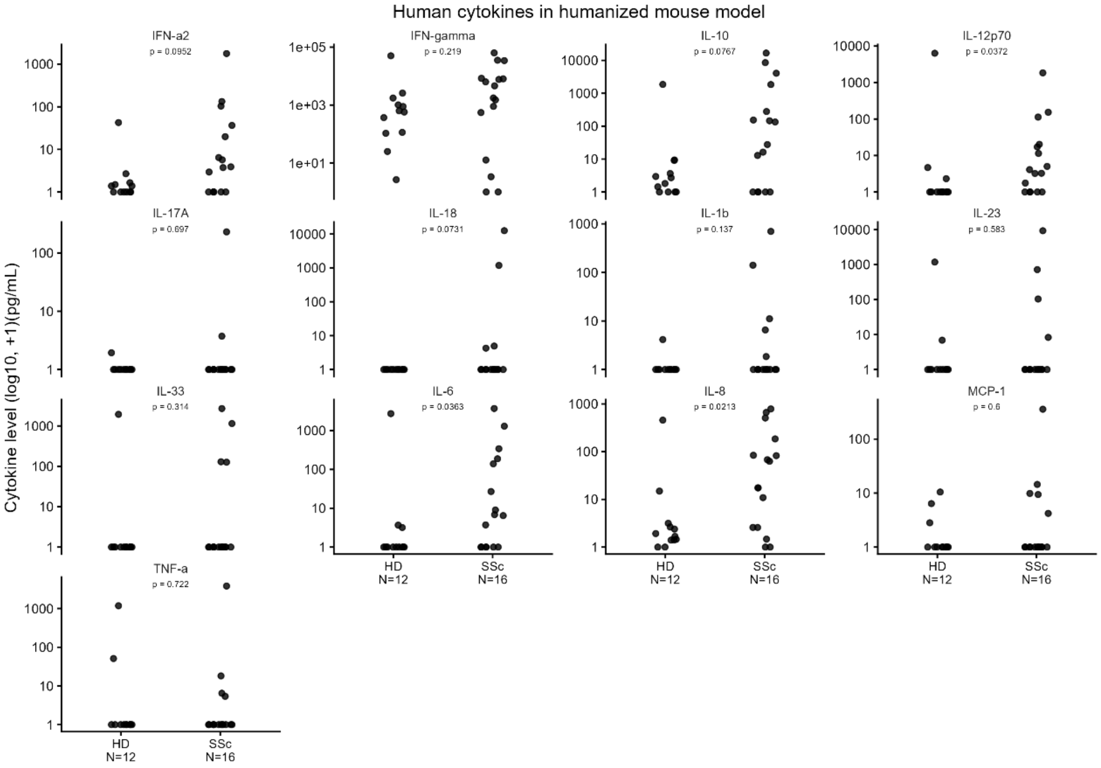

Panel 2

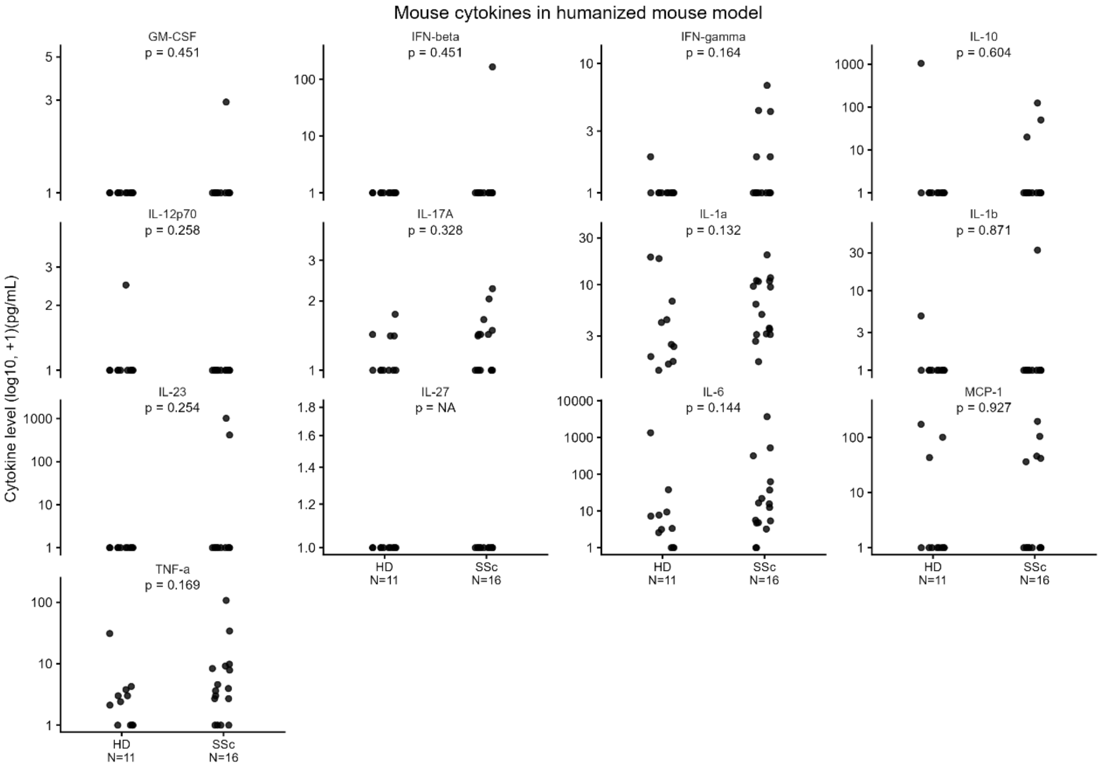

Figure S1. The graph with individual value plot for table 1. Panel 1: human cytokines in humanized mouse model; Panel 2: mouse cytokines in humanize mouse model. One sample is not available in the HD group of mouse cytokines determination. P values < 0.05 is considered as statistically significant difference.

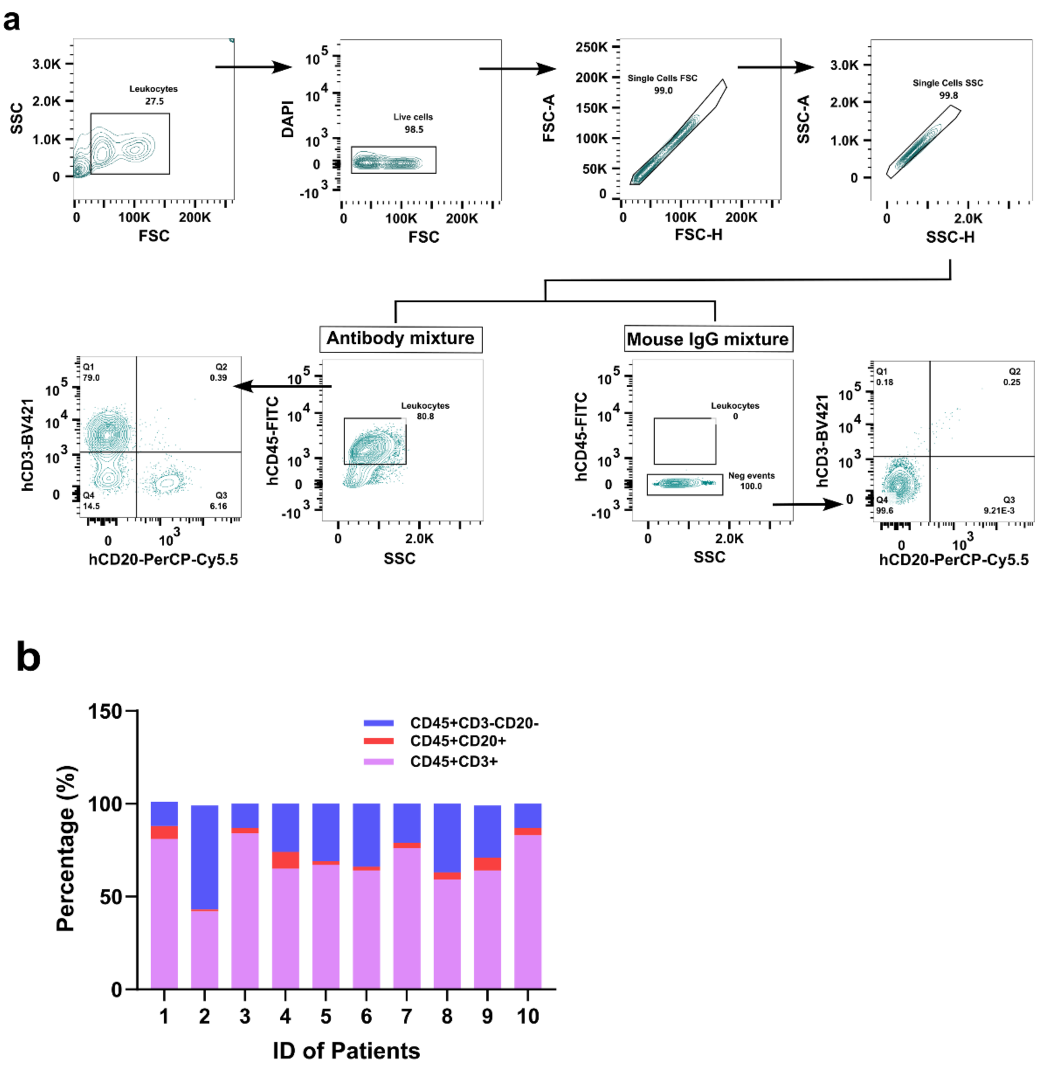

Figure S2. Cellular composition of PBMCs isolated from patients with SSc. Prior to engraftment into the humanized mouse model, different cell types within PBMCs from patients with SSc were identified using flow cytometry with antibodies targeting hCD45, hCD20, and hCD3(antibody mixture, corresponding isotype IgGs (mouse IgG mixture) were used as control. (A) Gating strategy employed for flow cytometry analysis of PBMCs. (B) Proportion of various immune cell types, including T cells (CD45+CD3+), B cells (CD45+CD20+), and CD45+CD3-CD20- leukocytes, are depicted in the bar graph.

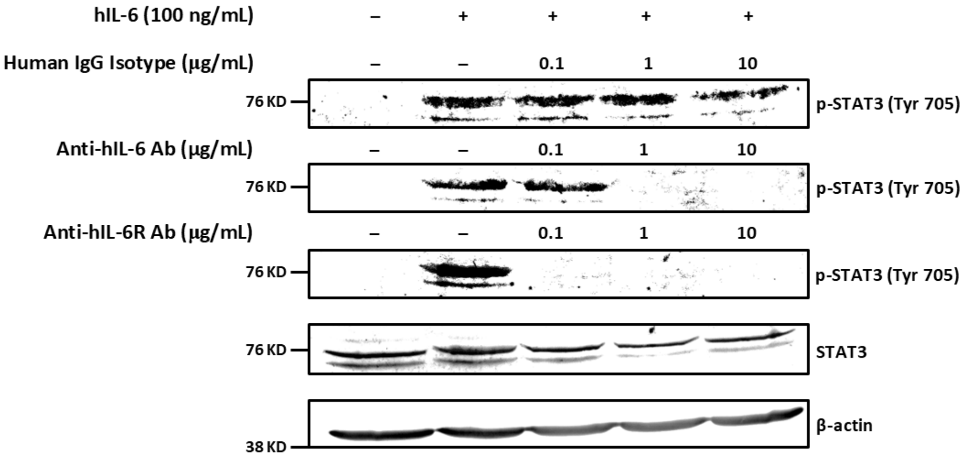

Figure S3. ***in vitro* effect of Tocilizumab and Siltuximab treatment on the expression of IL-6 signaling molecules of monocyte-like cells (U937).** U937 cells were pretreated with either human IgG1 isotype, anti-hIL-6R antibody (Tocilizumab), or anti-hIL-6 antibody (Siltuximab) at the specified concentrations, followed by stimulation with or without human IL-6 (100 ng/mL). After 10 minutes of stimulation, the cells were harvested and lysed for western blotting analysis. The following antibodies targeting IL-6 signaling activation markers were utilized: signal transducer and activator of transcription-3 (STAT3) and phosphorylated-STAT3 (p-STAT3). β-actin was employed as a loading control for the cell lysates.

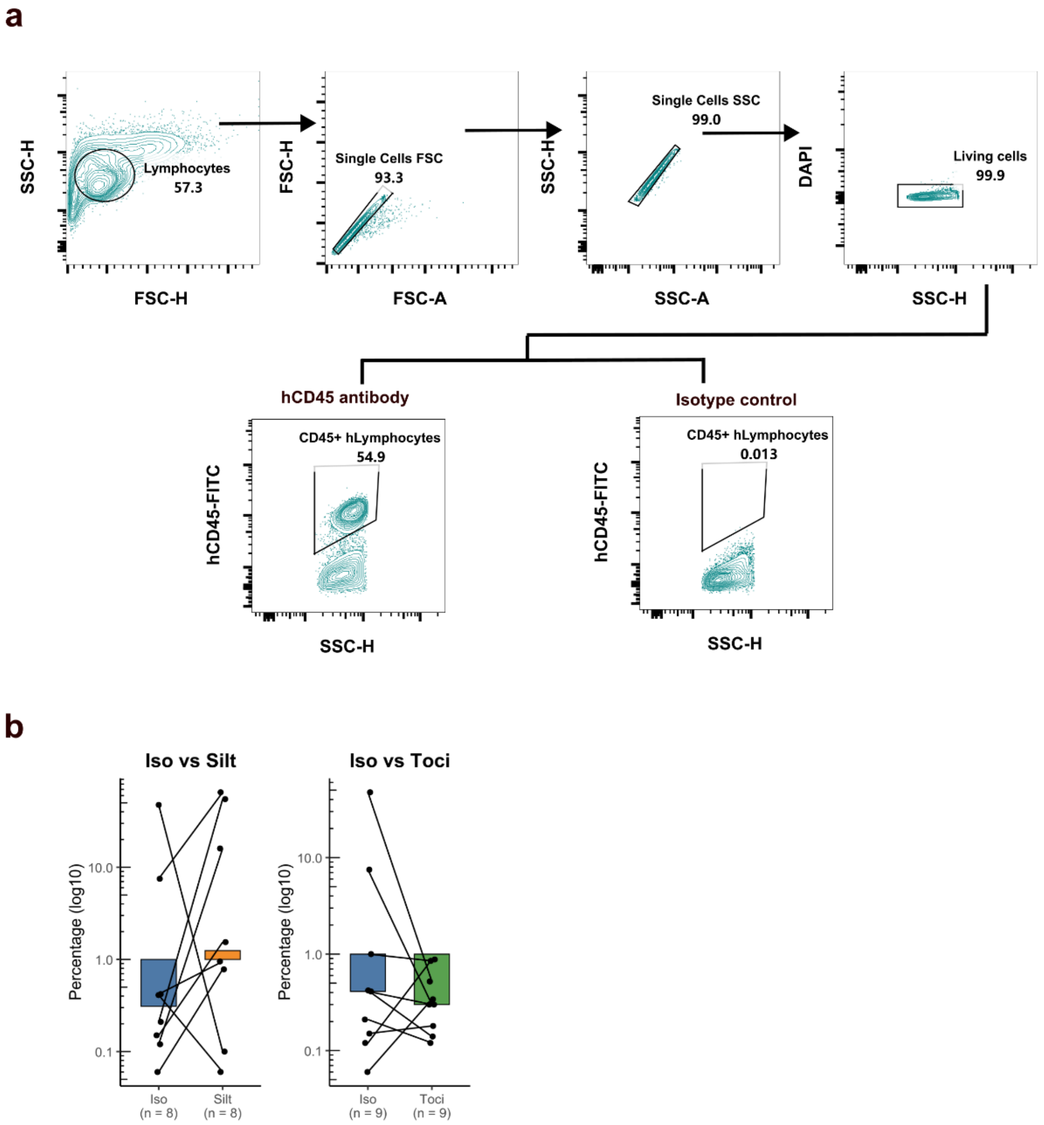

Figure S4. Validation of survival of human PBMCs in murine individuals. The gating strategies of flow cytometric analysis of splenocytes sample(a) at endpoint day of the experiment, the proportion of human CD45+ leukocytes are determined in the measurement. The percentage (log10) of human CD45+ cells out of total cell counts in spleen samples (b) are exhibited in bar plots. Only the paired samples are exhibited in figure (b). Due to death in the case of mouse, several samples were not available for FACS analysis, therefore the sample number is not always 10. Iso, isotype control; Silt, Siltuximab; Toci, Tocilizumab.

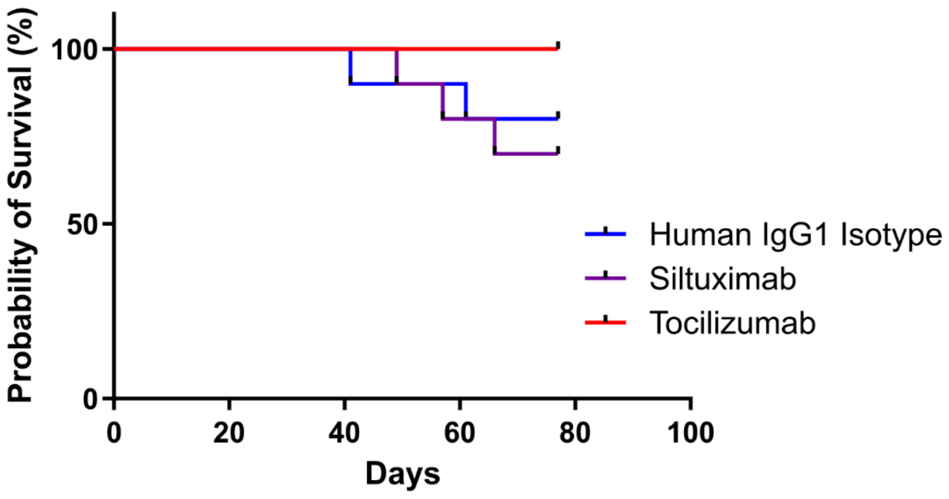

Figure S5. Kaplan-Meier survival analysis for the humanized mice. The eleven-week Kaplan-Meier estimates depict the cumulative probability of survival following the transfer of patients' PBMCs into three groups of mice. Each group comprised 10 mice.
